# Prevalence of risk phenotypes associated with CYP2C9*2, *3, and *VKORC1 c.-1639G>A* genetic polymorphisms in world populations: implications in clinical practice

**DOI:** 10.3389/fphar.2025.1597379

**Published:** 2025-09-19

**Authors:** Mohitosh Biswas, Murshadul Alam Murad, Maliheh Ershadian, Most. Sumaiya Khatun Kali, Maw Shin Sim, Baharudin Ibrahim, Chonlaphat Sukasem

**Affiliations:** ^1^ Department of Pharmacy, Faculty of Science, University of Rajshahi, Rajshahi, Bangladesh; ^2^ Division of Pharmacogenomics and Personalized Medicine, Department of Pathology, Faculty of Medicine Ramathibodi Hospital, Mahidol University, Bangkok, Thailand; ^3^ Laboratory for Pharmacogenomics, Somdech Phra Debaratana Medical Center (SDMC), Ramathibodi Hospital, Bangkok, Thailand; ^4^ Department of Pharmacy, Daffodil International University, Dhaka, Bangladesh; ^5^ Department of Pharmaceutical Life Sciences, Faculty of Pharmacy, Universiti Malaya, Kuala Lumpur, Malaysia; ^6^ Department of Clinical Pharmacy and Pharmacy Practice, Faculty of Pharmacy, Universiti Malaya, Kuala Lumpur, Malaysia; ^7^ Pharmacogenomics and Precision Medicine, The Preventive Genomics & Family Check-up Services Center, Bumrungrad International Hospital, Bangkok, Thailand; ^8^ Faculty of Pharmaceutical Sciences, Burapha University, Saensuk, Chonburi, Thailand

**Keywords:** genetic polymorphisms, *CYP2C9*, *VKORC1*, safety, efficacy, pharmacogenomics, precision medicine, clinical practice

## Abstract

**Background:**

The safety or efficacy of drugs may be affected by the genetic variability of *CYP2C9* or *VKORC1.* Patients may be at increased risk of drug-related toxicities, for example, bleeding events, if they carry CYP2C9*2 (rs1799853), CYP2C9*3 (rs1057910), or *VKORC1 c.-1639G>A* (rs9923231) genetic variants.

**Methods:**

The allele frequencies of CYP2C9*2, *3, and *VKORC1 c.-1639G>A* were obtained from the 1000 Genomes Project Phase III in line with Fort Lauderdale principles. Predictive risk phenotypes and correlations were assigned based on the carrying of characteristic allele carriers following international pharmacogenomics (PGx)-based dosing guidelines.

**Results:**

Intermediate and poor metabolizers were predicted to be risk phenotypes (17.8%; 95% CI 16.3%–19.3%) due to inheriting CYP2C9*2 and *3 genetic variants. These risk phenotypes were highest in European (35%; 95% CI 30.8%–39.2%), followed by South Asian (26.8%; 95% CI 22.9%–30.7%), American (25.9%; 95% CI 21.3%–30.5%), East Asian (6.7%; 95% CI 4.5%–8.9%), and African populations (2.1%; 95% CI 1%–3.2%). In addition, sensitive and highly sensitive responders were considered risk phenotypes (33.1%; 95% CI 31.3%–35%) when combining CYP2C9*2 and *3 variants with *VKORC1c.-1639G>A.* These risk phenotypes were most prevalent in East Asian (79.6%; 95% CI 76%–83.1%), followed by European (38.6%; 95% CI 34.3%–42.8%), American (30%; 95% CI 25.2%–34.8%), South Asian (25.2%; 95% CI 21.3%–29%), and African populations (1.2%; 95% CI 0.4%–2%), respectively. The prevalence of risk phenotypes in different ethnic groups was statistically significant (*p* < 0.05; 1.94 × 10^−175^, *χ* ^*2*^ test). According to clinical annotations in the PharmGKB, the safety or efficacy of at least 29 commonly prescribed drugs is impacted by the genetic polymorphisms of *CYP2C9*/*VKORC1 c.-1639G>A* to varying degrees. The PGx label information is available for at least 23 drugs, and the Clinical Pharmacogenetics Implementation Consortium (CPIC) has already recommended PGx-based dosing guidelines for at least 11 of these medications, based on the genetic variability of *CYP2C9*/*VKORC1 c.-1639G>A*.

**Conclusion:**

To enhance the safety of at least 11 clinically significant drugs, the current study found that approximately one-fifth of the global population is at risk based on the CYP2C9*2 and *3 genotypes. Additionally, approximately one-third of the population is at risk when considering the combination of *CYP2C9* and *VKORC1 c.-1639G>A* genotypes. Further studies are warranted to evaluate the clinical benefits of integrating PGx-based therapies in routine practice.

## Introduction

Genetic polymorphism refers to gene variations occurring at a frequency of 1% or higher in the human population (Brookes, 1999; Chiarella et al., 2023). Individual drug response is chiefly determined by the drug’s pharmacodynamic and pharmacokinetic features, which are directly or indirectly linked to the polymorphism in genes encoding the enzymes responsible for drug metabolism (Ahmed et al., 2016). To optimize both effectiveness and safety in treatment, it is advisable to employ precision medicine, which tailors patient care based on individual genetic traits. In this approach, particular attention is given to the polymorphisms of genes encoding cytochrome P450 (CYP) enzymes, as approximately 80% of phase-1 drug biotransformation is facilitated by a diverse array of CYP enzymes (Ashley, 2016; Collins and Varmus, 2015; Tornio and Backman, 2018; Zanger and Schwab, 2013; Zhou et al., 2017; Biswas, 2021). Of these, CYP2C9 is the predominantly expressed CYP2 isoform in the liver, making up ∼20% of the hepatic CYP proteins measured by mass spectrometry (Zhang et al., 2016). It is involved in the metabolism of a range of widely prescribed drugs such as coumarin anticoagulants, statins, non-steroidal anti-inflammatory drugs (NSAIDs), phenytoin, and sulfonylureas (Cooper-Dehoff et al., 2022; Daly et al., 2017; Johnson et al., 2017; Karnes et al., 2021; Relling and Klein, 2011; Yasar et al., 2003). *CYP2C9* is a highly polymorphic gene with at least 85 known variant alleles identified to date (Babaev et al., 2025). CYP2C9*2 (*p.R144C; rs1799853*) and CYP2C9*3 (*p.I359L; rs1057910*) are two well-studied genetic variants known to alter the safety and effectiveness of various medications. For instance, individuals carrying the CYP2C9*2 allele exhibit a 30%–40% reduction in CYP2C9 function, which leads to increased systemic exposure to fluvastatin. Those with the CYP2C9*3 allele experience a significantly more pronounced impact, with an approximately 80% reduction in CYP2C9 function (Cooper-Dehoff et al., 2022). Similarly, individuals inheriting one or two copies of CYP2C9*2 or *3 exhibited a greater risk of bleeding toxicity with warfarin therapy, as CYP2C9*2 and *3, respectively, impair 30%–40% and 80%–90% of the metabolism of S-warfarin (Lee et al., 2002; Mega et al., 2015). Due to the substantial impact of *CYP2C9* variants, the US Food and Drug Administration (FDA) and the European Medicines Agency (EMA) recommended genotyping for *CYP2C9* in the product characteristics summary or drug labels of 19 drugs (Zhou et al., 2023). The Clinical Pharmacogenetics Implementation Consortium (CPIC) also made similar recommendations, emphasizing the testing and adjusting dosages for several commonly prescribed drugs (Karnes et al., 2021; Johnson et al., 2017; Theken et al., 2020; Cooper-Dehoff et al., 2022).

On the other hand, *VKORC1* is situated on chromosome 16 and encodes the vitamin K epoxide reductase protein, which is associated with the metabolism of coumarin anticoagulants like warfarin (Gaikwad et al., 2014; Johnson et al., 2017). Among the several identified single-nucleotide polymorphisms (SNPs) in the *VKORC1* gene*, VKORC1 c.-1639G>A* (rs9923231) is specifically notable because of its impact on warfarin dosing by causing changes in the enzymatic activities of VKORC that significantly affects warfarin’s anticoagulant activity (Liu S. et al., 2021; Yang et al., 2010; Eriksson and Wadelius, 2012). *VKORC1 c.-1639G>A* alters the gene promoter activity, leading to lower warfarin doses being required for individuals inheriting allele A compared to people with the GG wild type (Panchenko et al., 2020; Ghafoor et al., 2022; Altawil and Youssef, 2023). Consequently, a reduction of warfarin dose by 70% in patients carrying the AA genotype was suggested by the Food and Drug Administration (FDA) in the 2007 drug label (Eriksson and Wadelius, 2012). Combinations of *CYP2C9* variants and *VKORC1 c.-1639G>A* have been used as the indication for choosing effective warfarin doses and managing the incidents of bleeding toxicity and have been extensively studied in many clinical studies (Yee et al., 2019; Vuorinen et al., 2021; Sridharan et al., 2021; Shukla et al., 2019; Liu J. et al., 2021; Dietz et al., 2020; Wang et al., 2024; Fahmi et al., 2024).

Previous studies exhibited substantial differences in the allele frequencies of *CYP2C9* and *VKORC1 c.-1639G>A* among various populations and ethnic groups (Sangkuhl et al., 2021; Nizamuddin et al., 2021; Gaikwad et al., 2013; Lund et al., 2012; Ma et al., 2012; Pourgholi et al., 2016; Vuorinen et al., 2021). Given the critical role in clinical outcomes, studies of the prevalence of *CYP2C9* and *VKORC1 c.-1639G>A* variants in global populations are therefore warranted. Only a few such studies were identified, but those did not include *VKORC1 c.-1639G>A,* and individuals within the same geographic group most commonly conformed to subpopulations (Céspedes-Garro et al., 2015; Sangkuhl et al., 2021; Sistonen et al., 2009; Zhou et al., 2023). No study has been identified to date that correlates the risk phenotypes with the number of clinically prescribed medications.

### Aims

Most genetic frequency studies commonly use subpopulations living within the same geographic area, which greatly limits the generalizability of the findings. We aimed to determine the predictive prevalence of CYP2C9*2, *3, and *VKORC1 c.-1639G>A* genetic polymorphisms on a global scale by utilizing the genetic data from the 1000 Genomes Project, comprising 2,504 participants from 26 different populations, to determine the predictive risk phenotypes. We also aimed to establish a correlation between the identified risk phenotypes and important commonly prescribed clinical drugs, whose safety and effectiveness may be affected by the risk phenotypes due to carrying genetic variants.

## Methods

### Study participants

This study analyzed genetic data for CYP2C9*2, *3, and *VKORC1 c.-1639G>A* from five different continental groups consisting of America (AMR), Africa (AFR), Europe (EUR), East Asia (EAS), and South Asia (SAS) (Auton et al., 2015; Sudmant et al., 2015). A total of 347 healthy individuals from four distinct ethnic groups (MXL = Mexican ancestry in Los Angeles, California; CLM = Colombian in Medellin, Colombia; PUR = Puerto Rican in Puerto Rico; and PEL = Peruvian in Lima, Peru) participated in the 1000 Genomes project for the continent of America. Similarly, across Europe, 503 healthy volunteers from five different ethnic groups (FIN = Finnish in Finland; CEU = Utah residents with Northern and Western European ancestry; GBR = British in England and Scotland; TSI = Tuscany in Italy; and IBS = Iberian populations in Spain) participated in the 1,000 Genome project. For the continent of Africa, seven ethnic groups (ASW = African ancestry in Southwest US; ACB = African Caribbean in Barbados; ESN = Esan in Nigeria; LWK = Luhya in Webuye, GWD = Gambian in Western Division, The Gambia; Kenya; YRI = Yoruba in Ibadan, Nigeria; and MSL = Mende in Sierra Leone) comprising 661 healthy volunteers were included in the 1,000 Genome project. Five different ethnic groups (GIH = Gujarati Indian in Houston; BEB, Bengali in Bangladesh; ITU = Indian Telugu in the United Kingdom; STU = Sri Lankan Tamil in the United Kingdom; and PJL = Punjabi in Lahore, Pakistan) containing 489 healthy individuals were included in the 1000 Genomes project for the continent of South Asia. For East Asia, 504 healthy volunteers from five ethnic groups (CHB = Han Chinese in Beijing; CDX = Chinese Dai in Xishuangbanna, China; CHS = Southern Han Chinese, China; KHV = Kinh in Ho Chi Minh City, Vietnam; and JPT = Japanese in Tokyo, Japan) participated in the 1000 Genomes project.

### Genetic data

Adhering to the Fort Lauderdale principles, the frequency of the genotypes and alleles of 2,504 participants of 26 distinct populations from five continental groups across the world carrying CYP2C9*1, CYP2C9*2 (rs1799853), CYP2C9*3 (rs1057910)*,* or *VKORC1 c.-1639G>A* (rs9923231) were extracted from 1000 Genomes Project Phase III. We then classified them into genotype groups based on carriage of different combinations of the aforementioned alleles. For instance, a participant carrying two copies of the CYP2C9*3 allele was assigned as a genotype of CYP2C9*3/*3. Likewise, other genotypes were assigned based on the characteristic alleles carried by the participants.

### Determination of predicted phenotypes and risk phenotypes

Three phenotype groups were assigned based on the genotypes as detailed in the Clinical Pharmacogenetics Implementation Consortium Guideline. Individuals with CYP2C9*1/*1 were classified as the normal metabolizer (NM) group. Similarly, individuals carrying CYP2C9*1/*2, CYP2C9*1/*3, and CYP2C9*2/*2 conformed to the intermediate metabolizer (IM) group, while individuals inheriting CYP2C9*2/*3 and CYP2C9*3/*3 were assigned as poor metabolizers (PM) (Karnes et al., 2021; Theken et al., 2020; Cooper-Dehoff et al., 2022).

For warfarin, three functional genotype groups were then assigned based on combinations of *CYP2C9* and *VKORC1 c.-1639G>A* genotypes that correspond to the categories of updated warfarin labels by the FDA. Individuals carrying CYP2C9*1/*1 + VKORC1 c.-1639G/G, CYP2C9*1/*1 + VKORC1 c.-1639A/G, or CYP2C9*1/*2 + VKORC1 c.-1639G/G were grouped as normal responders (NR). In contrast, the carriers of CYP2C9*1/*1 + VKORC1 c.-1639A/A, CYP2C9*1/*2 + VKORC1 c.-1639A/G, CYP2C9*1/*2 + VKORC1 c.-1639A/A, CYP2C9*1/*3 + VKORC1 c.-1639G/G, CYP2C9*1/*3 + VKORC1 c.-1639A/G, or CYP2C9*2/*2 + VKORC1 c.-1639G/G were grouped as sensitive responders (SR). Individuals with CYP2C9*1/*3 + VKORC1 c.-1639A/A, CYP2C9*2/*2 + VKORC1 c.-1639A/G, CYP2C9*2/*2 + VKORC1 c.-1639A/A, CYP2C9*2/*3 + VKORC1 c.-1639G/G, CYP2C9*2/*3 + VKORC1 c.-1639A/G, CYP2C9*2/*3 + VKORC1 c.-1639A/A, CYP2C9*3/*3 + VKORC1 c.-1639G/G, CYP2C9*3/*3 + VKORC1 c.-1639A/G, or CYP2C9*3/*3 + VKORC1 c.-1639A/A formed the highly sensitive responder (HSR) group (Mega et al., 2015). Table 1 sums up the categories.

**TABLE 1:** Predictive phenotypes based on the combined genotypes of CYP2C9*2, CYP2C9*3, and *VKORC1 c.-1639G>A* genetic variants.

Genetic variants	*CYP2C9*						
		*1/*1	*1/*2	*1/*3	*2/*2	*2/*3	*3/*3
*VKORC1 c.-1639G>A*	*G/G*	Normal responder	Normal responder	Sensitive responder	Sensitive responder	Highly sensitive responder	Highly sensitive responder
	*A/G*	Normal responder	Sensitive responder	Sensitive responder	Highly sensitive responder	Highly sensitive responder	Highly sensitive responder
	*A/A*	Sensitive responder	Sensitive responder	Highly sensitive responder	Highly sensitive responder	Highly sensitive responder	Highly sensitive responder

According to the CPIC dosing guidelines for several clinically significant drugs, the IM and PM groups of predicted phenotypes based on the *CYP2C9* genotype pose a safety risk. Therefore, these phenotypes (IM and PM) are considered risk phenotypes (Cooper-Dehoff et al., 2022; Karnes et al., 2021; Theken et al., 2020). The association between the presence of genetic variants CYP2C9*2, *3, and *VKORC1 c.-1639G>A* and the bleeding risk with warfarin therapy has been explored and established in several previous studies (Bazan et al., 2014; Bedewy et al., 2018; Biswas et al., 2018; Eriksson et al., 2016; Ferder et al., 2010; Gaikwad et al., 2013; Higashi et al., 2002; Limdi et al., 2008; Shukla et al., 2019; Vuorinen et al., 2021). A higher risk of major hemorrhages (up to 2–5-fold) has been observed among genetically sensitive and highly sensitive patients (Mega et al., 2015; Limdi et al., 2008; Yang et al., 2019). Therefore, they were the risk population in this study.

### Linking risk CYP2C9*2, *3, and *VKORC1 c.-1639G>A* phenotypes with the safety and effectiveness of medications

The clinical annotations and PGx label information of CYP2C9*2, *3, and *VKORC1 c.-1639G>A* for various clinically important drugs were sourced from internationally renowned pharmacogenomics working bodies, including the FDA-approved drug label, PharmGKB, the EMA-approved drug label, and the Health Canada Santé Canada (HCSC) approved drug label. All of the information was sourced from the PharmGKB website (Barbarino et al., 2018; Relling and Klein, 2011). The correlation between the safety or efficacy and the predicted risk phenotype was established using freely available pharmacogenomic-based CPIC dosing guidelines for various drugs (Karnes et al., 2021; Johnson et al., 2017; Cooper-Dehoff et al., 2022; Theken et al., 2020).

### Human ethics approval

For the current study, we gathered all the presented human genetic data from Phase III of the 1000 Genomes Project that adhered to Fort Lauderdale principles. The dictates for publishing any result utilizing data from the 1000 Genomes Project require no additional human ethics approval, as it has been previously published in another study (Auton et al., 2015; Sudmant et al., 2015).

### Statistical analysis

Data analyses were performed using descriptive statistics, and MS Excel was applied for both analysis and graphical representations. We utilized descriptive statistics to determine the frequency and risk profiles, and a chi-square test was employed to establish the statistical significance across the population. The findings are presented in a line chart.

### Validation of data analysis

All the genetic data included in the present study were sourced as raw data from the 1000 Genomes Project, which were then grouped into genotype groups by utilizing the COUNTIFS function in MS Excel, followed by descriptive analyses. Two researchers independently carried out all the analyses, and the corresponding author then double-checked all the analyzed data collected from the two researchers and corrected any anomalies accordingly.

## Results

### Prevalence of CYP2C9*2, *3, and *VKORC1 c.-1639G>A* alleles and associated genotypes in 26 populations

The allele frequency of CYP2C9*2 was 4.8% (95% CI 4%–5.6%) in the global populations that participated in the 1000 Genomes Project and varied among all the populations studied. The European population had the highest prevalence (12.4%; 95% CI 11.1%–13.7%), and the American population ranked second with a frequency of 9.9% (95% CI 8.8%–11.1%). The allele frequency was found to be progressively lower in the South Asian (3.5%; 95% CI 2.8%–4.2%), African (0.8%; 95% CI 0.5%–1.2%), and East Asian populations (0.1%; 95% CI 0%–0.2%). The allele frequency of CYP2C9*3 was 4.9% (95% CI 4%–5.7%) in the global populations that participated in the 1000 Genomes Project, where the South Asian population had the highest prevalence of CYP2C9*3 (10.9%; 95% CI 9.7%–12.2%). The European population had the second highest prevalency group with a frequency of 7.3% (95% CI 6.2%–8.3%), followed by the American (3.7%; 95% CI 3%–4.5%), East Asian (3.4%; 95% CI 2.7%–4.1%), and African (0.2%; 95% CI 0%–0.4%) populations, respectively.

Similar variability was observed for the allele frequency of *VKORC1 c.-1639G>A*. The prevalence of this variant allele was 35.6% (95% CI 33.7%–37.4%) in the global populations that participated in the 1000 Genomes Project. The highest prevalence was in the East Asian population with a frequency of 88.5% (95% CI 87.2%–89.7%), followed by American (41.1%; 95% CI 39.1%–43%), European (38.8%; 95% CI 36.9%–40.7%), South Asian (14.5%; 95% CI 13.1%–15.9%), and African (5.4%; 95% CI 4.6%–6.3%) populations. For the *A/G* type genotype, however, the American population exhibited the highest frequency of 47.6% (95% CI 42.3%–52.8%), closely followed by the European group with 46.1% (95% CI 41.8%–50.5%). The lowest frequency (10% 95% CI 7.7%–12.3%) was observed for the African population. South Asian and East Asian populations showed frequencies of 24.1% (95% CI 20.3%–27.9%) and 19.4% (95% CI 16.0%–22.9%), respectively. The *A/A* genotype was predominantly observed in the East Asian population, where its prevalence reached 78.8% (95% CI: 75.2%–82.3%). In the American population, it was found in 17.3% (95% CI: 13.3%–21.3%), making it the second-most common group, followed by the European population with a prevalence of 15.7% (95% CI: 12.5%–18.9%). South Asian and African populations had a low prevalence of 2.5% (95% CI 1.1%–3.8%) and 0.5% (95% CI 0%–1%), respectively.

The predictive genotypes were established using different combinations of the different variants of *CYP2C9,* as shown in Table 2. Participants inheriting two normal copies, that is, CYP2C9*1/*1, were the most prevalent (82.2%; 95% CI 80.7%–83.7%) in all populations. Individuals with a normal copy and a *3 allele (CYP2C9*1/*3) were the second most prevalent, with a global frequency of 8.5% (95% CI 7.4%–9.6%), followed by CYP2C9*1/*2 (7.7%; 95% CI 6.7%–8.7%), CYP2C9*2/*3 (0.8%; 95% CI 0.5%–1.1%), CYP2C9*2/*2 (0.6%; 95% CI 0.3%–0.9%), and CYP2C9*3/*3 (0.2%; 95% CI 0%–0.4%). Likewise, for *VKORC1 c.-1639G>A,* the average prevalence in all populations was the highest for *G/G* (50.9%; 95% CI 48.9%–52.9%), followed by *A/G* (27.1%; 95% CI 25.4%–28.8%) and *A/A* (22%; 95% CI 20.4%–23.6%).

**TABLE 2:** Frequency of genotypes associated with carrying either CYP2C9*2, CYP2C9*3, or *VKORC1 c.-1639G>A* genetic variants in the world populations participating in the 1000 Genomes Project.

Population	Genotype								
	*CYP2C9*						*VKORC1 c.-1639G>A*		
	*1/*1	*1/*2	*2/*2	*1/*3	*3/*3	*2/*3	*G/G*	*A/G*	*A/A*
AFR	97.9	1.7	0.0	0.5	0.0	0.0	89.6	10.0	0.5
AMR	74.1	17.9	0.6	6.6	0.0	0.9	35.2	47.6	17.3
EAS	93.3	0.2	0.0	6.3	0.2	0.0	1.8	19.4	78.8
SAS	73.2	5.3	0.2	19.4	0.6	1.2	73.4	24.1	2.5
EUR	65.0	18.5	2.2	12.1	0.2	2.0	38.2	46.1	15.7
All populations	**82.2**	**7.7**	**0.6**	**8.5**	**0.2**	**0.8**	**50.9**	**27.1**	**22.0**

Entries in bold represent the average prevalence in all populations.

### Prevalence of predicted phenotypes in 26 populations

**FIGURE 1:**
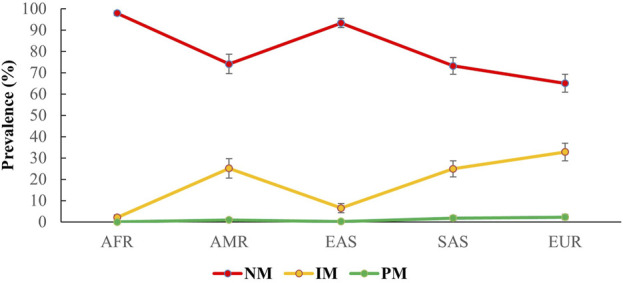
Predicted prevalence of different phenotypes for *CYP2C9* among different populations participating in the 1000 Genomes Project. The phenotypes were grouped on the basis of being a carrier of CYP2C9*1, *2, and *3 alleles following international guidelines. Here, AFR: Africans, AMR: Ad.Mix Americans, EAS: East Asians, SAS: South Asians, EUR: Europeans. NM, normal metabolizers; IM, intermediate metabolizers; PM, poor metabolizers.

As detailed in the methods section, predictive phenotype groups, such as NMs, IMs, and PMs, were assigned according to genotype, as shown in Figure 1. The NMs had an average prevalence of 82.2% (95% CI 80.7%–83.7%) for all 26 populations, and NM had the highest prevalence in the African population (97.9%; 95% CI 96.8%–99%), followed by East Asian (93.3%; 95% CI 91.1%–95.4%), American (74.1%; 95% CI 69.5%–78.7%), South Asian (73.2%; 95% CI 69.3%–77.1%), and European populations (65%; 95% CI 60.8%–69.2%). In contrast, the average prevalence of IMs in all populations was 16.8% (95% CI 15.3%–18.3%). Geographical group analysis showed the prevalence of IMs in European (32.8%; 95% CI 28.7%–36.9%), South Asian (24.9%; 95% CI 21.1%–28.8%), American (25.1%; 95% CI 20.5%–29.6%), East Asian (6.5% 95% CI 4.4%–8.7%) and African populations (2.1% 95% CI 1%–3.2%). Finally, PMs had an average prevalence of 1% (95% CI 0.6%–1.3%) for all populations, and geographical group analysis showed the prevalence of PMs in European (2.2%; 95% CI 0.9%–3.5%), South Asian (1.8%; 95% CI 0.6%–3%), American (0.9%; 95% CI 0%–1.8%) and East Asian populations (0.2%; 95% CI 0%–0.5%). PMs were not observed in the African population.

**FIGURE 2:**
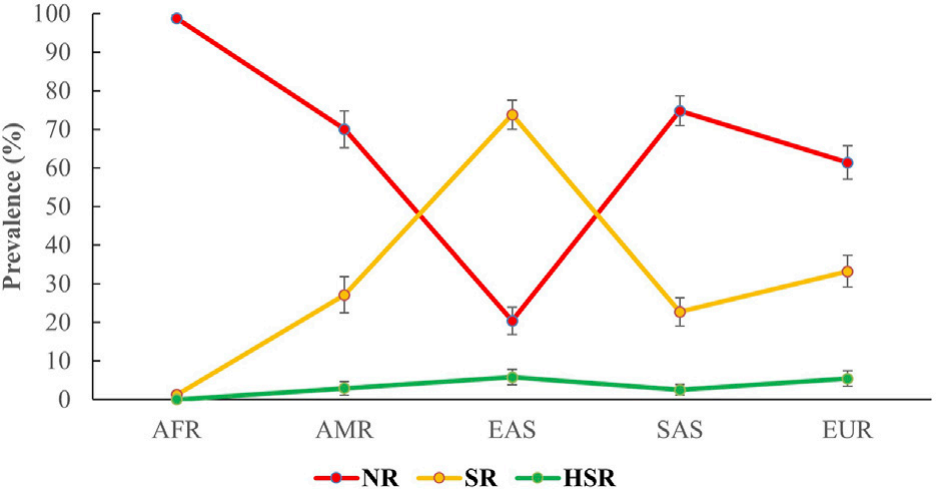
Predicted prevalence of different phenotypes of the combinations of *CYP2C9* and *VKORC1 c.-1639G>A* among different populations participating in the 1000 Genomes Project. The phenotypes were grouped on the basis of being a carrier of the variants of *CYP2C9* and *VKORC1 c.-1639G>A* alleles, following international guidelines. Here, AFR: Africans, AMR: Americans, EAS: East Asians, SAS: South Asians, EUR: Europeans. NR, normal responders; SR, sensitive responders; HRS, highly sensitive responders.

For the combination of *CYP2C9* and *VKORC1 c.-1639G>A*, we classified the predictive phenotypes into three distinguished groups, that is, NR, SR, and HSR, as described in the methods section. After grouping the combined genotypes into subsequent predictive phenotype groups, based on the geographical analysis, we observed the highest prevalence of NR in the African population (98.8%; 95% CI 98%–99.6%), sequentially followed by the South Asian (74.8%; 95% CI 71%–78.7%), American (70%; 95% CI 65.2%–74.8%), European (61.4%; 95% CI 57.2%–65.7%), and, finally, the East Asian population (20.4%; 95% CI 16.9%–24%). Interestingly, the East Asian population marked the highest prevalence (73.8%; 95% CI 70%–77.6%) of SR, while the lowest prevalence of SR was observed in the African population (1.2%; 95% CI 0.4%–2%). The prevalence of SR in European, American, and South Asian populations was 33.2% (95% CI 29.1%–37.3%), 27.1% (95% CI 22.4%–31.8%), and 22.7% (95% CI 19%–26.4%), respectively. Finally, for HSR, the highest frequency was observed in the East Asian population (5.8%; 95% CI 3.7%–7.8%), followed by the European (5.4%; 95% CI 3.4%–7.3%), American population (2.9%; 95% CI 1.1%–4.6%), and South Asian populations (2.5%; 95% CI 1.1%–3.8%). HSRs were not identified in the African population. The frequency of the phenotype groups for different populations is shown in Figure 2.

### Prevalence of risk phenotypes in 26 populations

As outlined in the method portion, the risk phenotypes of *CYP2C9* were determined by the frequency of IM and PM. Among the 2,504 participants of the 1000 Genomes Project, we identified approximately one-fifth (17.8%; 95% CI 16.3%–19.3%) of the *CYP2C9* risk phenotypes. Geographical analysis revealed variability in the frequency of risk phenotypes among different populations. The European group had the highest prevalence, with a frequency of 35% (95% CI 30.8%–39.2%). The South Asian population followed with a frequency of 26.8% (95% CI 22.9%–30.7%), while the American population had a prevalence of 25.9% (95% CI 21.3%–30.5%). The East Asian population showed a frequency of 6.7% (95% CI 4.5%–8.9%), and the African population had the lowest prevalence at 2.1% (95% CI 1.1%–3.2%). For the combined *CYP2C9* and *VKORC1 c.-1639G>A* genotypes, SR and HSR comprised the risk phenotypes, which we found to be approximately one-third (33.1%; 95% CI 31.3%–35%) of the 2,504 individuals partaking in the 1000 Genomes Project. Geographical group analysis revealed that the East Asian population is in the lead with a frequency of 79.6% (95% CI 76%–83.1%) of the risk phenotypes, and the African population, with only 1.2% (95% CI 0.4%–2%) frequency, was the group with the lowest prevalence. The European, American, and South Asian populations had frequencies of 38.6% (95% CI 34.3%–42.8%), 30% (95% CI 25.2%–34.8%), and 25.2% (95% CI 21.3%–29%), respectively. The prevalence of risk phenotypes in different ethnic groups was statistically significant (*p < 0.05;* 1.94 × 10^−175^, *χ* ^*2*^ test).

### Linking *CYP2C9* and *VKORC1 c.-1639G>A* risk phenotypes with the safety or effectiveness of drugs

According to clinical annotations of the PharmGKB, the safety or efficacy of at least 29 commonly prescribed clinical drugs is impacted by the genetic polymorphism of *CYP2C9* in varying degrees, as depicted through the evidence levels ranging from Level 4 (preliminary) to Level 1A (Pharmgkb, 2025a). In total, 11 of them, that is, warfarin, celecoxib, flurbiprofen, fluvastatin, ibuprofen, lornoxicam, meloxicam, phenytoin, piroxicam, siponimod, and tenoxicam, were said to have strong evidence (Level 1A). One drug (acenocoumarol) had Level 1B evidence; at least 14 drugs had a low level of evidence (Level 3) and at least three drugs had Level 4 (unsupported) evidence for interfering with the safety or effectiveness due to the presence of a CYP2C9*2 or *3 allele. In contrast, *VKORC1 c.-1639G>A* was associated with the safety of at least three drugs, that is, warfarin, acenocoumarol, and phenprocoumon, all having Level 1A evidence, as described in clinical annotations of the PharmGKB (Pharmgkb, 2025b).

PGx label information from the FDA, EMA, HCSC, Pharmaceuticals and Medical Devices Agency (PMDA), and Swissmedic for at least 23 drugs mentioned the genetic variability of *CYP2C9* *.* The FDA’s table of Pharmacogenomic Biomarkers in Drug Label was the source for most of the PGx label information. However, the PGx label information of at least five drugs, that is, avatrombopag, etrasimod, prasugrel, rimegepant, and Siponimod, was identified from the FDA, EMA, and HCSC. The label emphasizes the requirement of PGx testing for siponimod alongside the label of “Actionable PGx” for 15 drugs, stating the need for dose adjustment or alternative therapy for individuals with certain problematic genotypes or risk phenotypes if known, although it does not recommend genotype testing prior to the use of these drugs (Pharmgkb, 2025). On the other hand, “No Clinical PGx” offers specific genetic information and its impact on drug concentration, metabolism, and side effects, but no specific clinical guideline is provided. Three drugs, that is, abrocitinib, rimegepant, and prasugrel, were identified as being in the “No Clinical PGx” category due to *CYP2C9* variants. As for *VKORC1 c.-1639G>A,* warfarin is the only drug with drug-level annotation. Both the FDA and HCSC deemed the association as actionable PGx. Detailed lists of the drugs can be found in Table 3.

**TABLE 3:** The PharmGKB drug labels for the *CYP2C9* and *VKORC1 c.-1639G>A* genetic variants.

Drugs	FDA	EMA	HCSC	PMDA	Swissmedic	Genetic variants
Avatrombopag	Informative PGx	Actionable PGx	Actionable PGx	-	-	CYP2C9*2, CYP2C9*3
Celecoxib	Actionable PGx	-	Actionable PGx	Actionable PGx	Actionable PGx	CYP2C9*2, CYP2C9*3
Dronabinol	Actionable PGx	-	-	-	-	CYP2C9*2, CYP2C9*3
Erdafitinib	Actionable PGx	-	Actionable PGx	-	-	CYP2C9*3
Etrasimod	Informative PGx	Actionable PGx	Informative PGx	-	-	CYP2C9*2, CYP2C9*3
Flibanserin	Informative PGx	-	Informative PGx	-	-	CYP2C9*2, CYP2C9*3
Flurbiprofen	Actionable PGx	-	Actionable PGx	-	-	CYP2C9*2, CYP2C9*3
Fosphenytoin	Actionable PGx	-	Informative PGx	-	-	CYP2C9*2, CYP2C9*3
Glyburide	-	-	Actionable PGx	-	-	CYP2C9*2, CYP2C9*3
Lesinurad	Actionable PGx	Actionable PGx	-	-	Informative PGx	CYP2C9*1, CYP2C9*3
Meloxicam	Informative PGx	-	-	-	-	CYP2C9*2, CYP2C9*3
Nateglinide	Informative PGx	-	-	-	-	CYP2C9*2, CYP2C9*3
Phenytoin	Actionable PGx	-	Informative PGx	-	Actionable PGx	CYP2C9*2, CYP2C9*3
Piroxicam	Actionable PGx	-	-	-	Actionable PGx	CYP2C9*2, CYP2C9*3
Prasugrel	-	-	-	-	Informative PGx	*CYP2C9*
Siponimod	Testing required	Testing required	Testing required	-	-	CYP2C9*1, CYP2C9*2, CYP2C9*3
Warfarin	Actionable PGx	-	Actionable PGx	-	-	CYP2C9*1, CYP2C9*2, CYP2C9*3, *rs9923231*
Glimepiride	-	-	Actionable PGx	-	-	CYP2C9*2, CYP2C9*3
Losartan	-	-	-	-	Actionable PGx	CYP2C9*3
Acenocoumarol	-	-	-	-	Actionable PGx	CYP2C9*2, CYP2C9*3
Brivaracetam	-	-	-	-	Informative PGx	CYP2C9*2, CYP2C9*3

Here, FDA, Food and Drug Administration; EMA, European Medicines Agency; HCSC, Health Canada Santé Canada; PMDA, Pharmaceuticals and Medical Devices Agency; PGx, pharmacogenomics.

To date, the CPIC has provided PGx-based dosing guidelines for 11 drugs based on *CYP2C9* variability (Cooper-Dehoff et al., 2022; Johnson et al., 2017; Karnes et al., 2021; Theken et al., 2020). In contrast, for warfarin, a detailed guideline based on the ancestry and the presence of genetic variants in the *CYP2C9, VKORC1,* and *CYP4F2* genes, as well as *rs12777823,* has been recommended by the CPIC (Johnson et al., 2017). The guidelines have been summarized in Tables 4, 5 which strongly advise the careful handling of the IM and PM phenotypes for the optimization of the safety or effectiveness of many clinically significant drugs. For example, alternative therapies that are not impacted by the genetic variability of *CYP2C9,* are not metabolized by CYP2C9, or are metabolized by CYP2C9 but have a short half-life should be considered instead of piroxicam and tenoxicam, as the phenotypes correspond to *CYP2C9* IM and PM.

**TABLE 4:** The CPIC dosing guidelines for drugs based on the *CYP2C9* genetic polymorphism.

Drugs	CYP2C9 phenotype	Recommendations	Classification of recommendations	References
Celecoxib, flurbiprofen, ibuprofen, lornoxicam	IM (AS-1)	CPIC recommends the initiation of the therapy with the lowest recommended starting dose in CYP2C9 IM with an AS of 1.	Moderate	Theken et al. (2020)
	PM	Initiate the therapy with 25%–50% of the lowest recommended dose.	Moderate	Theken et al. (2020)
Fosphenytoin, phenytoin	IM (AS-1)	IMs with an AS of 1 may opt for reduced doses as increased plasma concentration increases the probability of toxicities.	Moderate	Karnes et al. (2021)
	PM	PMs may opt for reduced doses as increased plasma concentration increases the probability of toxicities.	Strong	Karnes et al. (2021)
Fluvastatin	IM	Doses >40 mg should be avoided in IMs. Alternative statins are suggested if higher doses are crucial to obtain the desired efficacy.	Moderate	Cooper-Dehoff et al. (2022)
	PM	Doses >20 mg should be avoided in PMs. Alternative statins are suggested if higher doses are crucial to obtain the desired efficacy.	Moderate	Cooper-Dehoff et al. (2022)
Meloxicam	IM (AS-1)	Initiate therapy with 50% of the lowest recommended dose in CYP2C9 IMs with the AS of 1.	Moderate	Theken et al. (2020)
	PM	As a significant prolonged half-life is observed with meloxicam in PMs, alternative therapies are recommended.	Moderate	Theken et al. (2020)
Piroxicam, tenoxicam	PM/IM	Alternative therapies that are not metabolized by CYP2C9 or are not notably affected by CYP2C9 variants *in vivo*, or an NSAID that is metabolized by CYP2C9 and possesses a shorter half-life, should be recommended for PMs and IMs.	Moderate	Theken et al. (2020)

Here, IM, intermediate metabolizers; PM, poor metabolizers; AS, activity score.

**TABLE 5:** The CPIC dosing guidelines for warfarin based on the *CYP2C9* and *VKORC1 c.-1639G>A* genetic polymorphisms.

Drugs	*CYP2C9* and *VKORC1 c.-1639G>A* combined phenotype	Recommendations	Classification of recommendations	References
Warfarin	HSR, SR	For non-African individuals carrying CYP2C9*2, *3, and *VKORC1 c.-1639G>A,* CPIC recommends dosing based on the validated PGx algorithms.	Strong	Johnson (2017)
		For African individuals carrying CYP2C9*2, *3, and *VKORC1 c.-1639G>A*, CPIC recommends dosing based on the validated PGx algorithms, and a reduction of the calculated dose by 15%–30% is recommended for the carriers of CYP2C9*5, *6, *8, or *11 variant alleles.	Moderate	

Here, SR, sensitive responders; HSR, highly sensitive responders.

## Discussion

We found that almost one-fifth (17.8%; 95% CI 16.3%–19.3%) of the participants of the 1000 Genomes Project are at an increased risk of drug-related toxicities of several clinically significant drugs that are affected by the genetic variability of *CYP2C9*. Similarly, the combination of *CYP2C9* and *VKORC1 c.-1639G>A* genetic polymorphisms imposes risk on 33.1% (95% CI 31.3%–35%) of all the individuals who participated in the 1000 Genomes Project if they are taking warfarin therapy. This study also observed that South Asian and European populations are particularly at high risk for *CYP2C9* variability, and East Asian and European populations are the most affected by the combined genotype of *CYP2C9* and *VKORC1 c.-1639G>A*. The insightful findings can be viewed as new information, and further studies may be conducted, keeping these populations in mind, to design therapies to optimize the safety of the drugs of concern.

The global prevalence data observed in the current study for CYP2C9*2 and *3 from 26 different ethnic groups conforming to five geographical populations were in line with the previously published literature and provide supporting evidence for them (Céspedes-Garro et al., 2015; Sangkuhl et al., 2021; Sistonen et al., 2009). Likewise, the frequency of *VKORC1 c.-1639G>A* variants among different world populations also complied with the frequency found in several studies focusing on specific populations (Dandara et al., 2011; Buzoianu et al., 2012; Gaikwad et al., 2014; Meckley et al., 2008).

Overall, the estimated risk profile reported provides a strong base for the initial assessment of the global population. However, genetic data from the Middle East and North Africa (MENA) region and the Oceania continents are absent in the 1000 Genomes Project, and this may impact the overall estimation of the global risk population. In addition, phenoconversion (the mismatch between the genotype-oriented drug metabolism prediction and the true individual phenotype due to the comorbidity, comedication, or other non-genetic factors) should also be considered. Phenoconversion may affect genotypes in different ways (Klomp et al., 2020). For example, investigation of concomitant treatment with a CYP2C9 substrate (flurbiprofen) and a CYP2C9 inhibitor (fluconazole) revealed that with a 200 mg dose of fluconazole, *CYP2C9* NMs are converted to IMs, while the IMs convert to PMs. With the 400 mg dose of fluconazole, both NMs and IMs convert to PMs (Kumar et al., 2008). Because these factors may limit the generalizability of the result presented, more specific and large-scale assessments are required to reach a comprehensive understanding of the risk population.

Several international PGx working groups are providing clinical guidelines to translate PGx data into clinical practice. By compiling the approved PGx information together and categorizing it into various evidence levels, the PharmGKB is now emerging as a significant source of PGx information. For example, the PharmGKB offered varying degrees of evidence of clinical association for at least 29 drugs for CYP2C9*2 and *3 and at least three drugs for *VKORC1 c.-1639G>A* variants. These associations are then translated into clinical practice as “Testing Required,” “Testing Recommended,” “Actionable PGx,” “Informative PGx,” “No Clinical PGx,” and “Criteria Not Met.” The FDA is at the forefront in offering PGx information compared to other global regulatory authorities. Around three-fourths of the PGx information of *CYP2C9* (73.9%) was provided by the FDA alone. However, this information also existed in different combinations from other PGx working bodies like the EMA, HCSC, PMDA, and Swissmedic.

The CPIC has thus far provided dosing guidelines for at least 10 drugs based on the genetic polymorphism of *CYP2C9,* particularly considering the presence of CYP2C9*2 and CYP2C9*3. The CPIC also provided a detailed dosing guideline for warfarin, taking the combination of *CYP2C9* and *VKORC1 c.-1639G>A* into account. Adhering to the consensus standardization for characterizing phenotypes based on carriers of certain genotypes, as detailed in the CPIC, this study is uniquely constructed to identify phenotypes based on the CPIC dosing guidelines. No study thus far has explored the prevalence of the variants of *CYP2C9* and *VKORC1 c.-1639G>A* together on a global scale to categorize them in predictive phenotype groups, thereby associating them with the safety of several clinical drugs and available dosing guidelines.

The findings of the frequency of predictive risk phenotype for *CYP2C9* variants (17.8%; 95% CI 16.3%–19.3%) and the combination of *CYP2C9* and *VKORC1 c.-1639G>A* variants (33.1%; 95% CI 31.3%–35%) among 26 different world populations represented by 2,504 participants of the 1000 Genomes Project thus give uniqueness to the study. This further reinforces the need for the implementation of PGx-based therapy for the drugs in concern among the risk phenotypes. For example, meloxicam, with a longer half-life (15–20 h), imposes the risk of increased exposure in impaired meloxicam metabolizers. Therefore, the CPIC recommended alternative therapy for *CYP2C9* poor metabolizers and in IM with an AS of 1, initiating the therapy with 50% of the lowest recommended dose to avoid toxicities (Theken et al., 2020).

It should also be emphasized that the genetic variability of *CYP2C9* may not always cause all of the pharmacokinetic variation. Alongside *CYP2C9,* other genetic factors may also contribute to the safety or effectiveness of the same drug. For example, fluvastatin is impacted by *SLCO1B1* and *CYP2C9* variants, phenytoin metabolism is affected by the variants of *CYP2C19, CYP1A1*, and *EPHX1*, and, on the other hand, the safety of warfarin therapy is dependent on the genetic variabilities of *CYP2C9, VKORC1, CYP4F2*, and rs12777823 (Cooper-Dehoff et al., 2022; Johnson et al., 2017; Karnes et al., 2021; Hung et al., 2012; Wilke et al., 2005). Therefore, developing pharmacogenomic polygenic response scores (PGxRS) can serve as a valuable source for predicting the occurrence of side effects associated with certain drugs, especially when multiple genetic polymorphisms are expected to alter the safety or effectiveness profile of the drug.

Although several international PGx working bodies are working to translate the PGx information into clinical practice, insufficient evidence and incomplete correlation of genetic variation and phenotypic outcomes remain the most significant setbacks for the widespread application of PGx-driven precision medicine into clinical practice. These authorities continue to provide updates on PGx-based dosing; however, the application is still limited. The present study aimed to bridge the existing knowledge gap by providing robust scientific evidence for the authorities and stakeholders to make more informed decisions, with an emphasis on PGx integration for the drugs with a high association with genetic variabilities to optimize the safety of certain drugs, as reported in this study.

### Limitations

Because it is a predictive analysis, variation in the prevalence of the estimated risk phenotype may arise because the CYP2C9 and VKORC1 enzyme function was not directly measured; rather, various combinations of genotype were employed. The absence of genetic data from the Middle East and North Africa (MENA) region and the Oceania continents in the 1000 Genomes Project limited the global population coverage of the study.

### Future directions

As the present study established the association of the safety of at least 11 drugs with CYP2C9*2, *3, and *VKORC1 c.-1639G>A* genetic polymorphisms, future studies should emphasize large-scale longitudinal PGx-based therapy to assess toxicities in real-world clinical settings. In addition, the drugs categorized into different evidence levels on the PharmGKB should be further explored to gather sufficient evidence for conclusive decisions.

## Conclusion

For optimizing the safety of at least 11 clinically significant drugs, the present study identified that approximately one-fifth and one-third of the global population are at risk based on the *CYP2C9* and the combination of *CYP2C9* and *VKORC1 c.-1639G>A* genotypes, respectively. Further large-scale longitudinal studies on different ethnicities are warranted to evaluate the clinical benefits of integrating PGx-based therapies in routine practice and achieve precision medicine.

## Data Availability

The datasets presented in this study can be found in online repositories. The names of the repository/repositories and accession number(s) can be found in the article/supplementary material.
